# Ethyl 2-(5-phenyl-1,3,4-oxadiazol-2-ylsulfan­yl)acetate

**DOI:** 10.1107/S1600536808007125

**Published:** 2008-03-20

**Authors:** Muhammad Zareef, Rashid Iqbal, Muhammad Arfan, Masood Parvez

**Affiliations:** a Department of Chemistry, Quaid-i-Azam University, Islamabad 45320, Pakistan; b Department of Chemistry, The University of Calgary, 2500 University Drive NW, Calgary, Alberta, Canada T2N 1N4

## Abstract

The title mol­ecule, C_12_H_12_N_2_O_3_S, is composed of two individually planar units, *viz*. 5-phenyl-1,3,4-oxadiazol-2-yl-sulfanyl and ethyl acetate, which are oriented at almost right angles [80.07 (8)°] with respect to each other. The structure is stabilized by weak inter­molecular C—H⋯O and C—H⋯N hydrogen bonds. The phenyl and oxadiazole rings show π–π stacking inter­actions [centroid–centroid distance = 3.846 (2) Å] and there is also a short π-inter­action between the carbonyl O atom and the oxadiazole ring [the distance from this O atom to the centroid of the oxadiazole ring is 3.156 (2) Å].

## Related literature

For related literature, see: Cao *et al.* (2002 ▶); Iqbal *et al.* (2007 ▶); Kadi *et al.* (2007 ▶); Mir & Siddiqui (1970 ▶); Zareef *et al.* (2006 ▶, 2007 ▶).
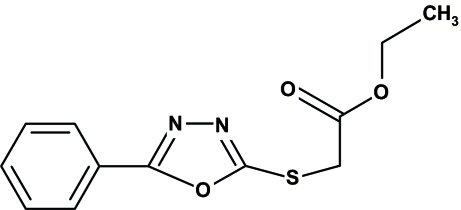


## Experimental

### 

#### Crystal data

C_12_H_12_N_2_O_3_S
*M* *_r_* = 264.30
Monoclinic, 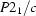

*a* = 8.777 (3) Å
*b* = 11.008 (5) Å
*c* = 13.177 (6) Å
β = 103.59 (3)°
*V* = 1237.5 (9) Å^3^
*Z* = 4
Mo *K*α radiation
μ = 0.26 mm^−1^
*T* = 173 (2) K
0.16 × 0.10 × 0.08 mm

#### Data collection

Nonius KappaCCD diffractometer
Absorption correction: multi-scan (*SORTAV*; Blessing, 1997 ▶) *T*_min_ = 0.959, *T*_max_ = 0.979
5263 measured reflections
2820 independent reflections
1943 reflections with *I* > 2σ(*I*)
*R*_int_ = 0.046

#### Refinement

*R*[*F* ^2^ > 2σ(*F* ^2^)] = 0.045
*wR*(*F* ^2^) = 0.117
*S* = 1.02
2820 reflections
165 parameters
H-atom parameters constrained
Δρ_max_ = 0.25 e Å^−3^
Δρ_min_ = −0.27 e Å^−3^

### 

Data collection: *COLLECT* (Hooft, 1998 ▶); cell refinement: *DENZO* (Otwinowski & Minor, 1997 ▶); data reduction: *SCALEPACK* (Otwinowski & Minor, 1997 ▶); program(s) used to solve structure: *SAPI91* (Fan, 1991 ▶); program(s) used to refine structure: *SHELXL97* (Sheldrick, 2008 ▶); molecular graphics: *ORTEP-3 for Windows* (Farrugia, 1997 ▶); software used to prepare material for publication: *SHELXL97*.

## Supplementary Material

Crystal structure: contains datablocks global, I. DOI: 10.1107/S1600536808007125/fb2092sup1.cif

Structure factors: contains datablocks I. DOI: 10.1107/S1600536808007125/fb2092Isup2.hkl

Additional supplementary materials:  crystallographic information; 3D view; checkCIF report

## Figures and Tables

**Table 1:** Hydrogen-bond geometry (Å, °)

*D*—H⋯*A*	*D*—H	H⋯*A*	*D*⋯*A*	*D*—H⋯*A*
C4—H4⋯O2^i^	0.95	2.51	3.268 (3)	137
C9—H9*B*⋯N1^ii^	0.99	2.38	3.293 (3)	153

Symmetry codes: (i) 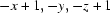; (ii) 
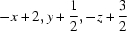
.

**Fig. 1.**
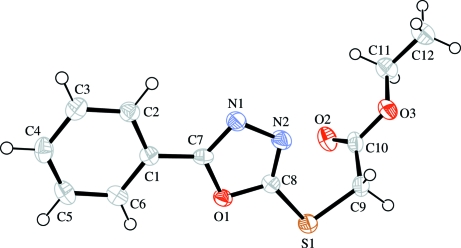
ORTEP-3 (Farrugia, 1997) drawing of the title molecule with displacement ellipsoids plotted at 50% probability level.

## supplementary crystallographic information

### Comment

Substituted-1,3,4-oxadiazole derivatives are of significant interest due to their chemotherapeutic effects (Kadi *et al.*, 2007; Zareef *et al.*, 2006; Zareef *et al.*, 2007; Cao *et al.*, 2002). Based on the known structures of the 2,5-disubstituted-1,3,4-oxadiazoles with diverse biological activities and their derivatives, we have designed and synthesized several new derivatives of 1,3,4-oxadiazole (Zareef *et al.*, 2007). In this paper, we report the structure of one of these compounds.

The structure of the title compound (Fig. 1) is composed of two essentially planar moieties, C1—C8/N1/N2/O1/S1 and C9—C12/O2/O3 the least-square planes of which are inclined at 80.07 (8)°; the maximum deviations from the respective least square planes are: O1 = 0.037 (2) and C11 = 0.048 (2) Å. The structure is stabilized by two intermolecular interactions C4—H4···O2 and C9—H9B···N1 (Table 1). The shortest distance between the centroids of the phenyl and the oxadiazole rings of the adjacent molecules is 3.846 (2) Å which indicates the existence of π-π stacking interactions. In addition, there is a π-interaction between the carbonyl O-atom and the oxadiazole ring. (The distance from this O atom to the centroid of the oxadiazole ring is 3.156 (2) Å). The bond distances and angles in the title compound are in agreement with the corresponding ones reported in the similar structure of Ethyl 2-({5-[2-(benzoylamino)phenyl]-1,3,4-oxadiazol-2-yl}sulfanyl)acetate (Iqbal *et al.*, 2007).

### Experimental

The title compound was prepared according to the procedure reported in the literature (Zareef *et al.*, 2006; Mir & Siddiqui, 1970). To a solution of benzoic acid hydrazide (50 mmol) in ethanol (150 ml) was added carbon disulfide (55 mmol), followed by the addition of KOH (50 mmol) dissolved in 25 ml of water. The reaction mixture was stirred and subjected to reflux for 19 h. After reaction completion, excess ethanol was distilled off. The crude solid obtained was dissolved in water (50 ml) and acidified with 4 N HCl to pH 2–3. The product was filtered, washed with water and recrystallized from aqueous ethanol (20–30%). The resulting 5-phenyl-2-mercapto-1,3,4-oxadiazole (20 mmol) was dissolved in saturated aqueous sodium hydrogencarbonate solution while stirring. The required ethylbromoacetate (20 mmol) in absolute ethanol (10 ml) was added and the reaction mixture was stirred for 7 h at 325–335 K. After reaction completion, the resulting solid was filtered off, washed with water and recrystallized from aqueous ethanol (60%) (Yield = 75%; m.p. = 344–345 K). Prismatic crystals suitable for crystallographic study were grown from ethanol solution by slow evaporation at room temperature.

### Refinement

Though all the H atoms could be distinguished in the difference Fouries map the H-atoms were situated at the geometrically idealized positions and refined in riding-model approximation with the following constraints: aryl, methylene and methyl C—H distances were set to 0.95, 0.99 and 0.98 Å, respectively; in all these instances *U*_iso_(H) = 1.2 *U*_eq_(C).

### Crystal data

**Table:**

C_12_H_12_N_2_O_3_S	*F*_000_ = 552
*M**_r_* = 264.30	*D*_x_ = 1.419 Mg m^−^^3^
Monoclinic, *P*2_1_/*c*	Melting point = 344–345 K
Hall symbol: -P 2ybc	Mo *K*α radiation λ = 0.71073 Å
*a* = 8.777 (3) Å	Cell parameters from 5263 reflections
*b* = 11.008 (5) Å	θ = 3.7–27.5º
*c* = 13.177 (6) Å	µ = 0.26 mm^−^^1^
β = 103.59 (3)º	*T* = 173 (2) K
*V* = 1237.5 (9) Å^3^	Prism, colourless
*Z* = 4	0.16 × 0.10 × 0.08 mm

### Data collection

**Table:**

Nonius KappaCCD diffractometer	2820 independent reflections
Radiation source: fine-focus sealed tube	1943 reflections with *I* > 2σ(*I*)
Monochromator: graphite	*R*_int_ = 0.046
*T* = 173(2) K	θ_max_ = 27.5º
ω and φ scans	θ_min_ = 3.7º
Absorption correction: multi-scan(SORTAV; Blessing, 1997)	*h* = −11→11
*T*_min_ = 0.959, *T*_max_ = 0.979	*k* = −13→14
5263 measured reflections	*l* = −17→17

### Refinement

**Table:**

Refinement on *F*^2^	Secondary atom site location: difference Fourier map
Least-squares matrix: full	Hydrogen site location: difference Fourier map
*R*[*F*^2^ > 2σ(*F*^2^)] = 0.045	H-atom parameters constrained
*wR*(*F*^2^) = 0.117	*w* = 1/[σ^2^(*F*_o_^2^) + (0.058*P*)^2^ + 0.1*P*] where *P* = (*F*_o_^2^ + 2*F*_c_^2^)/3
*S* = 1.02	(Δ/σ)_max_ = 0.001
2820 reflections	Δρ_max_ = 0.25 e Å^−^^3^
165 parameters	Δρ_min_ = −0.27 e Å^−^^3^
47 constraints	Extinction correction: SHELXL97 (Sheldrick, 2008), Fc^*^=kFc[1+0.001xFc^2^λ^3^/sin(2θ)]^-1/4^
Primary atom site location: structure-invariant direct methods	Extinction coefficient: 0.021 (3)

### Special details

**Table:**

Geometry. All e.s.d.'s (except the e.s.d. in the dihedral angle between two l.s. planes) are estimated using the full covariance matrix. The cell e.s.d.'s are taken into account individually in the estimation of e.s.d.'s in distances, angles and torsion angles; correlations between e.s.d.'s in cell parameters are only used when they are defined by crystal symmetry. An approximate (isotropic) treatment of cell e.s.d.'s is used for estimating e.s.d.'s involving l.s. planes.

### Fractional atomic coordinates and isotropic or equivalent isotropic displacement parameters (Å^2^)

**Table:**

	*x*	*y*	*z*	*U*_iso_*/*U*_eq_	
S1	0.76489 (6)	0.36024 (5)	0.69782 (4)	0.0336 (2)	
O1	0.67313 (14)	0.20172 (11)	0.54296 (10)	0.0264 (3)	
O2	0.86894 (16)	0.19182 (14)	0.88791 (12)	0.0412 (4)	
O3	1.12555 (15)	0.22664 (13)	0.90282 (11)	0.0354 (4)	
N1	0.87361 (18)	0.07562 (15)	0.55872 (13)	0.0307 (4)	
N2	0.91309 (18)	0.16477 (15)	0.63754 (13)	0.0319 (4)	
C1	0.6404 (2)	0.03494 (16)	0.41660 (14)	0.0245 (4)	
C2	0.7088 (2)	−0.06503 (17)	0.37915 (15)	0.0283 (5)	
H2	0.8131	−0.0882	0.4116	0.034*	
C3	0.6246 (2)	−0.13002 (18)	0.29499 (16)	0.0339 (5)	
H3	0.6717	−0.1972	0.2690	0.041*	
C4	0.4712 (2)	−0.09762 (19)	0.24806 (16)	0.0335 (5)	
H4	0.4130	−0.1429	0.1905	0.040*	
C5	0.4040 (2)	0.0005 (2)	0.28557 (16)	0.0342 (5)	
H5	0.2991	0.0225	0.2534	0.041*	
C6	0.4870 (2)	0.06791 (19)	0.36949 (15)	0.0291 (5)	
H6	0.4397	0.1357	0.3945	0.035*	
C7	0.7333 (2)	0.10069 (16)	0.50595 (15)	0.0245 (4)	
C8	0.7925 (2)	0.23515 (17)	0.62486 (15)	0.0266 (4)	
C9	0.9529 (2)	0.35400 (18)	0.78938 (17)	0.0337 (5)	
H9A	1.0358	0.3499	0.7498	0.040*	
H9B	0.9684	0.4302	0.8304	0.040*	
C10	0.9730 (2)	0.24772 (19)	0.86414 (16)	0.0311 (5)	
C11	1.1623 (2)	0.1290 (2)	0.97896 (17)	0.0392 (6)	
H11A	1.1105	0.0528	0.9492	0.047*	
H11B	1.1252	0.1498	1.0422	0.047*	
C12	1.3366 (2)	0.1135 (2)	1.00597 (17)	0.0421 (6)	
H12A	1.3655	0.0466	1.0558	0.051*	
H12B	1.3864	0.1887	1.0373	0.051*	
H12C	1.3722	0.0952	0.9425	0.051*	

### Atomic displacement parameters (Å^2^)

**Table:**

	*U*^11^	*U*^22^	*U*^33^	*U*^12^	*U*^13^	*U*^23^
S1	0.0300 (3)	0.0316 (3)	0.0369 (3)	0.0048 (2)	0.0031 (2)	−0.0069 (2)
O1	0.0232 (7)	0.0275 (7)	0.0273 (7)	0.0048 (6)	0.0037 (5)	−0.0008 (6)
O2	0.0280 (7)	0.0554 (10)	0.0392 (9)	−0.0054 (7)	0.0056 (7)	−0.0009 (8)
O3	0.0235 (7)	0.0397 (8)	0.0407 (9)	0.0003 (6)	0.0026 (6)	0.0013 (7)
N1	0.0292 (8)	0.0333 (10)	0.0278 (9)	0.0061 (7)	0.0031 (7)	−0.0055 (8)
N2	0.0279 (9)	0.0354 (10)	0.0308 (10)	0.0070 (8)	0.0038 (7)	−0.0050 (8)
C1	0.0265 (9)	0.0261 (10)	0.0219 (10)	−0.0015 (8)	0.0074 (8)	0.0045 (8)
C2	0.0275 (10)	0.0286 (10)	0.0288 (11)	0.0000 (9)	0.0066 (8)	0.0020 (9)
C3	0.0403 (12)	0.0296 (11)	0.0327 (12)	−0.0022 (10)	0.0103 (9)	−0.0023 (9)
C4	0.0349 (11)	0.0373 (12)	0.0278 (11)	−0.0128 (10)	0.0061 (9)	−0.0010 (9)
C5	0.0250 (10)	0.0473 (13)	0.0286 (11)	−0.0036 (10)	0.0031 (9)	0.0057 (10)
C6	0.0265 (10)	0.0354 (11)	0.0264 (11)	0.0028 (9)	0.0085 (8)	0.0044 (9)
C7	0.0241 (9)	0.0235 (10)	0.0278 (10)	0.0056 (8)	0.0101 (8)	0.0034 (8)
C8	0.0243 (9)	0.0283 (10)	0.0267 (10)	0.0011 (9)	0.0050 (8)	0.0005 (8)
C9	0.0269 (10)	0.0336 (11)	0.0382 (12)	−0.0033 (9)	0.0027 (9)	−0.0088 (9)
C10	0.0241 (10)	0.0381 (12)	0.0300 (11)	−0.0027 (9)	0.0040 (8)	−0.0111 (9)
C11	0.0353 (11)	0.0437 (14)	0.0387 (13)	0.0012 (10)	0.0089 (10)	0.0031 (10)
C12	0.0356 (12)	0.0534 (15)	0.0360 (13)	0.0066 (11)	0.0056 (10)	0.0033 (11)

### Geometric parameters (Å, °)

**Table:**

S1—C8	1.729 (2)	C3—H3	0.9500
S1—C9	1.802 (2)	C4—C5	1.377 (3)
O1—C8	1.366 (2)	C4—H4	0.9500
O1—C7	1.369 (2)	C5—C6	1.387 (3)
O2—C10	1.202 (2)	C5—H5	0.9500
O3—C10	1.336 (2)	C6—H6	0.9500
O3—C11	1.454 (3)	C9—C10	1.513 (3)
N1—C7	1.294 (2)	C9—H9A	0.9900
N1—N2	1.412 (2)	C9—H9B	0.9900
N2—C8	1.291 (2)	C11—C12	1.497 (3)
C1—C6	1.392 (2)	C11—H11A	0.9900
C1—C2	1.398 (3)	C11—H11B	0.9900
C1—C7	1.457 (3)	C12—H12A	0.9800
C2—C3	1.379 (3)	C12—H12B	0.9800
C2—H2	0.9500	C12—H12C	0.9800
C3—C4	1.390 (3)		
			
C8—S1—C9	96.66 (9)	O1—C7—C1	120.13 (15)
C8—O1—C7	102.24 (13)	N2—C8—O1	113.20 (17)
C10—O3—C11	115.56 (16)	N2—C8—S1	128.61 (15)
C7—N1—N2	106.58 (15)	O1—C8—S1	118.18 (13)
C8—N2—N1	105.66 (15)	C10—C9—S1	114.43 (14)
C6—C1—C2	119.91 (17)	C10—C9—H9A	108.7
C6—C1—C7	121.99 (18)	S1—C9—H9A	108.7
C2—C1—C7	118.10 (16)	C10—C9—H9B	108.7
C3—C2—C1	119.90 (18)	S1—C9—H9B	108.7
C3—C2—H2	120.0	H9A—C9—H9B	107.6
C1—C2—H2	120.0	O2—C10—O3	124.49 (19)
C2—C3—C4	120.3 (2)	O2—C10—C9	125.87 (18)
C2—C3—H3	119.8	O3—C10—C9	109.62 (17)
C4—C3—H3	119.8	O3—C11—C12	107.26 (18)
C5—C4—C3	119.56 (18)	O3—C11—H11A	110.3
C5—C4—H4	120.2	C12—C11—H11A	110.3
C3—C4—H4	120.2	O3—C11—H11B	110.3
C4—C5—C6	121.14 (18)	C12—C11—H11B	110.3
C4—C5—H5	119.4	H11A—C11—H11B	108.5
C6—C5—H5	119.4	C11—C12—H12A	109.5
C5—C6—C1	119.2 (2)	C11—C12—H12B	109.5
C5—C6—H6	120.4	H12A—C12—H12B	109.5
C1—C6—H6	120.4	C11—C12—H12C	109.5
N1—C7—O1	112.32 (16)	H12A—C12—H12C	109.5
N1—C7—C1	127.55 (18)	H12B—C12—H12C	109.5
			
C7—N1—N2—C8	0.1 (2)	C6—C1—C7—O1	−3.2 (3)
C6—C1—C2—C3	0.6 (3)	C2—C1—C7—O1	177.68 (16)
C7—C1—C2—C3	179.73 (18)	N1—N2—C8—O1	−0.3 (2)
C1—C2—C3—C4	−0.9 (3)	N1—N2—C8—S1	178.66 (15)
C2—C3—C4—C5	0.5 (3)	C7—O1—C8—N2	0.4 (2)
C3—C4—C5—C6	0.1 (3)	C7—O1—C8—S1	−178.68 (14)
C4—C5—C6—C1	−0.3 (3)	C9—S1—C8—N2	0.1 (2)
C2—C1—C6—C5	0.0 (3)	C9—S1—C8—O1	179.01 (15)
C7—C1—C6—C5	−179.09 (18)	C8—S1—C9—C10	−69.82 (17)
N2—N1—C7—O1	0.2 (2)	C11—O3—C10—O2	−0.3 (3)
N2—N1—C7—C1	−179.84 (18)	C11—O3—C10—C9	177.96 (16)
C8—O1—C7—N1	−0.4 (2)	S1—C9—C10—O2	−22.7 (3)
C8—O1—C7—C1	179.66 (16)	S1—C9—C10—O3	159.06 (14)
C6—C1—C7—N1	176.80 (19)	C10—O3—C11—C12	175.61 (18)
C2—C1—C7—N1	−2.3 (3)		

### Hydrogen-bond geometry (Å, °)

**Table:**

*D*—H···*A*	*D*—H	H···*A*	*D*···*A*	*D*—H···*A*
C4—H4···O2^i^	0.95	2.51	3.268 (3)	137
C9—H9B···N1^ii^	0.99	2.38	3.293 (3)	153

Symmetry codes: (i) −*x*+1, −*y*, −*z*+1; (ii) −*x*+2, *y*+1/2, −*z*+3/2.
